# Extensions of the natural approach to refinements and generalizations of some trigonometric inequalities

**DOI:** 10.1186/s13662-018-1545-7

**Published:** 2018-03-14

**Authors:** Branko Malešević, Tatjana Lutovac, Marija Rašajski, Cristinel Mortici

**Affiliations:** 1 School of Electrical Engineering, University of Belgrade, Belgrade, Serbia; 2 Valahia University of Târgovişte, Târgovişte, Romania; 3 Academy of Romanian Scientists, Bucharest, Romania; 4 University Politehnica of Bucharest, Bucharest, Romania

**Keywords:** 33B10, 26D05, Sharpening, Generalization, Wilker–Cusa–Huygens’s inequalities, Sine and cosine functions, Leibniz’s theorem for alternating series

## Abstract

In this paper we propose a new method for sharpening and refinements of some trigonometric inequalities. We apply these ideas to some inequalities of Wilker–Cusa–Huygens type.

## Introduction

Inequalities involving trigonometric functions are used in many applications in various fields of mathematics such as difference equations and inequalities [1], theory of stability, theory of approximations, etc. A method called *the natural approach*, proposed by Mortici in [2], uses the idea of comparing functions to their corresponding Taylor polynomials. This method has been successfully applied to prove and approximate a wide category of trigonometric inequalities [3, 4].

In this paper we extend the ideas of the natural approach by comparing and replacing functions with their corresponding power series. In particular, we focus on the results of Mortici in [2] related to Wilker–Cusa–Huygens’s inequalities and give generalizations and refinements of the inequalities stated in Theorems 1, 2, 3, 4, 5, and 6 in that paper. They are cited below.

### Statement 1

([2], Theorem 1)

*For every* $0< x<\pi /2$, *we have*

$$ - \frac{x^{4}}{15}< \cos {x} - \biggl( \frac{\sin {x}}{x} \biggr) ^{ 3} < - \frac{x^{4}}{15} + \frac{23x^{6}}{1890}. $$

### Statement 2

([2], Theorem 2)

*For every* $0< x<\pi /2$, *we have*

$$ - \frac{1}{180}x^{4}< \frac{\sin {x}}{x} - \frac{ \cos {x} + 2}{3}< - \frac{1}{180}x^{4} + \frac{1}{3780}x ^{6}. $$

### Statement 3

([2], Theorem 3)

*For every* $0< x<\pi /2$, *we have*

$$ 3 + \biggl( \frac{3}{20}x^{4} - \frac{3}{140}x^{6} \biggr) \frac{1}{ \cos {x}}< 2 \frac{\sin {x}}{x} + \frac{\tan x}{x}< 3 + \frac{3}{20} \frac{x ^{4}}{\cos {x}}. $$

### Statement 4

([2], Theorem 4)

*For every* $0< x<\pi /2$, *we have*

$$ 2 + \biggl( \frac{8 x^{4}}{45}- \frac{8 x^{6}}{105} \biggr) \frac{1}{ \cos {x}}< \biggl( \frac{\sin {x}}{x} \biggr) ^{ 2}+ \frac{ \tan {x}}{x}< 2 + \frac{8 x^{4}}{45}\frac{1}{\cos {x}} . $$

### Statement 5

([2], Theorem 5)

*For every* $0< x<\pi /2$, *we have*

$$ \biggl( \frac{x}{\sin {x}} \biggr) ^{ 2} + \frac{x}{\tan {x}} > 2 + \frac{2}{45}x^{4}. $$

### Statement 6

([2], Theorem 6)

*For every* $0< x<\pi /2$, *we have*

$$ 3 \frac{x}{\sin {x}} + \cos {x} > 4 + \frac{1}{10}x^{4} + \frac{1}{210}x^{6}. $$

## Preliminaries

First, let us recall some of the well-known power series expansions that will be used in our proofs.

For $x \in R$, the following power series expansions hold:

1 $$ \sin x = \sum_{k=0}^{\infty }{(-1)^{k} \frac{1}{(2k+1)!} x^{2k+1}},\qquad \cos x = \sum _{k=0}^{\infty }(-1)^{k}{\frac{1}{ (2k)!} x^{2k}}. $$

Also, according to [5], for $x \in R$, we have the following power series expansions:

2 $$ \cos ^{3} x = \frac{1}{4}\sum _{k=1}^{\infty }{(-1)^{k}\frac{3^{2k} + 3 }{ (2k)!} x^{2k}} $$

and

3 $$ \sin^{3} x= \frac{1}{4} \sum _{k=1}^{\infty }{(-1)^{k+1} \frac{3^{2k+1}-3}{(2k+1)!}x^{2k+1}}. $$

For $x \in (0,\frac{\pi }{2} )$, according to [5], the following series expansions hold:

4 $$\begin{aligned}& \operatorname{cosec} (x) = \frac{1}{x} + \sum _{k = 1}^{ \infty } \frac{\vert \boldsymbol{B}_{2k}\vert (2^{2k} - 2)}{(2k)!}{x^{2k - 1}}, \end{aligned}$$

5 $$\begin{aligned}& \operatorname{cosec}^{2}(x) = \frac{1}{x^{2}} +\sum _{k = 1}^{ \infty }\frac{\vert \boldsymbol{B}_{2k} \vert (2k - 1)4^{k}}{(2k)!}x^{2k - 2}, \end{aligned}$$

and

6 $$ \operatorname{cotan}(x) = \frac{1}{x} -\sum _{k = 1}^{ \infty } \frac{\vert \boldsymbol{B}_{2k}\vert 4^{k}}{(2k)!}x^{2k - 1}, $$

where $\boldsymbol{B}_{i}$ are Bernoulli’s numbers.

### Theorem WD

([6], Theorem 2)

*Suppose that* $f(x)$ *is a real function on* $(a,b)$, *and that* *n* *is a positive integer such that* $f ^{(k)}(a+)$, $f^{(k)}(b-)$ ($k \in \{0,1,2, \ldots ,n\} $) *exist*.

(i) *Supposing that* $(-1)^{(n)} f^{(n)}(x)$ *is increasing on* $(a,b)$, *then for all* $x \in (a,b)$ *the following inequality holds*:
  7 $$\begin{aligned} &\sum_{k=0}^{n-1} \frac{f^{(k)}(b - )}{k!} (x-b)^{k}+ \frac{1}{(a-b)^{n}} \Biggl( f(a + ) - \sum _{k=0}^{n-1}\frac{(a-b)^{k}f^{(k)}(b - )}{k!} \Biggr) (x-b)^{n} \\ &\quad < f(x)< \sum_{k=0}^{n}{ \frac{f^{(k)}(b - )}{k!}(x-b)^{k}}. \end{aligned}$$
  *Furthermore*, *if* $(-1)^{n} f^{(n)}(x)$ *is decreasing on* $(a,b)$, *then the reversed inequality of* (7) *holds*.
(ii) *Supposing that* $f^{(n)}(x)$ *is increasing on* $(a,b)$, *then for all* $x \in (a,b)$ *the following inequality holds*:
  8 $$\begin{aligned} &\sum_{k=0}^{n-1} \frac{f^{(k)}(a+ )}{k!} (x-a)^{k}+\frac{1}{(b-a)^{n}} \Biggl(f(b-)-\sum _{k=0}^{n-1}\frac{(b-a)^{k} f^{(k)}(a +)}{k!}\Biggr) (x-a)^{n} \\ &\quad >f(x)>\sum_{k=0}^{n} \frac{f^{(k)}(a + )}{k!}(x-a)^{k}. \end{aligned}$$

*Furthermore*, *if* $f^{(n)}(x)$ *is decreasing on* $(a,b)$, *then the reversed inequality of* (8) *holds*.

Let us mention that an interesting application of Theorem WD is given in [7], see also [8].

## Main results

We need the following theorem for the proofs of Theorems 1, 2, 3, and 4.

### Proposition 1

*Let the series* $f(x)= \sum_{k=1}^{\infty }(-1)^{k+1} A(k) x^{2k}$ *converge for* $x \in (0,c)$, $c \in R^{+} $. *Suppose that the following statements are true*:

(i) *If* $c < 1$, *then the sequence* $\{ A(k) \} _{k \in N}$ *is a positive decreasing sequence that converges to* 0.
(ii) *If* $c \geq 1$, *then the sequence* $\{ A(k) \} _{k \in N}$ *is a positive sequence*, $\lim_{k\rightarrow +\infty } c^{2k}A(k) = 0$ *and* $A(k) > c^{2} A(k+1)$ *for* $k \geq 1$.

*Then*, *for all* $x \in (0, c)$ *and for all* $n \in N$ *and* $m \in N$, *we have*

9 $$ \sum_{k=1}^{2n}{(-1)^{k+1} A(k) x^{2k}} < f(x) < \sum_{k=1}^{2n+1}{(-1)^{k+1} A(k) x^{2k}} $$

*and*

10 $$ \Biggl\vert f(x) -\sum_{k=1}^{m}{(-1)^{k} A(k) x^{2k}} \Biggr\vert < A(m+1) x^{2m+2}< c^{2m+2} A(m+1). $$

### Proof

Suppose that $c < 1$. Then, for every $x \in (0,c)$, the positive sequence $\{ A(k) x^{2k} \} _{k \in N}$ decreases monotonically and $\lim_{k\rightarrow \infty } A(k) x^{2k} =0$. Thus, assertions (9) and (10) immediately follow from Leibniz’s theorem for the alternating series.

Suppose now that $c \geq 1$. We have

$$\begin{aligned} f(x)&=\sum_{k=1}^{\infty }(-1)^{k+1} A(k) x^{2k} \\ &= \sum_{k=1}^{\infty }(-1)^{k+1} A(k) c^{2k} \biggl( \frac{x}{c} \biggr)^{2k}. \end{aligned}$$

Let us introduce the substitution $t = \frac{x}{c}$ in the previous power series and consider the series

11 $$ \sum_{k=1}^{\infty }(-1)^{k+1} A(k) c^{2k} t^{2k} \quad \mbox{for } t \in (0,1). $$

For the assumption $A(k) > c^{2}A(k+1)$, we have

$$ A(k) > c^{2} A(k+1)\quad \Longleftrightarrow \quad A(k)c^{2k} > c ^{2k+2} A(k+1) \quad \mbox{for } k \geq 1. $$

Hence, we conclude that for every $t \in (0,1)$ power series (11) satisfies Leibniz’s theorem for the alternating series, and for all $n, m \in N$ we have

12 $$ \sum_{k=1}^{2n}{(-1)^{k+1} A(k) c^{2k} t^{2k}} < \sum_{k=1} ^{\infty }(-1)^{k+1} A(k) c^{2k} t^{2k} < \sum _{k=1}^{2n+1}{(-1)^{k+1} A(k) c^{2k} t^{2k}} $$

and

13 $$\begin{aligned} \Biggl\vert \sum_{k=1}^{\infty }(-1)^{k+1} A(k) c^{2k} t^{2k}-\sum_{k=1}^{m}{(-1)^{k+1} A(k) c^{2k} t^{2k}} \Biggr\vert &< A(m+1) c^{2m+2} t^{2m+2} \\ &< A(m+1) c^{2m+2}. \end{aligned}$$

Returning the variable $x= t c$ to (12) and (13) gives the assertions of proposition. □

### Refinements of the inequalities in Statement 1

We propose the following improvement and generalization of Statement 1.

#### Theorem 1

(i) *For every* $x \in ( 0, \frac{\pi}{2})$ *and every* $n \in N$, *we have*
  14 $$ \sum_{k=2}^{2n} (-1)^{k} A(k) x^{2k}< \cos x - \biggl(\frac{\sin x}{x} \biggr) ^{ 3}< \sum_{k=2}^{2n+1}(-1)^{k} A(k) x^{2k}, $$
  *where*
  $$ A(k) = \frac{3^{2k+3}-32k^{3}-96k^{2}-88k-27}{4(2k+3)!}. $$
(ii) *For every* $x \in ( 0, \frac{\pi }{2})$ *and every* $m \in N$, *we have the following error estimation*:
  15 $$ \Biggl\vert \cos x- \biggl(\frac{\sin x}{x} \biggr) ^{ 3}-\sum _{k=1}^{m}{(-1)^{k}A(k) x^{2k}} \Biggr\vert < A(m+1) x^{2m+2}. $$

#### Examples

Let $x\in( 0, \frac{\pi }{2}) $.

For $n=1$, we get Statement 1.

For $n>1$, we have the following new results:

- Taking $n=2$ in (14) gives
  $$\begin{aligned} \frac{1}{15}x^{4} + \frac{23}{1890}x^{6} - \frac{41}{37\text{,}800}x^{8}& < \cos x - \biggl(\frac{\sin x}{x}\biggr) ^{3} \\ &< - \frac{1}{15} x^{4} + \frac{23}{1890} x^{6} - \frac{41}{37\text{,}800}x^{8} + \frac{53}{831\text{,}600} x^{10}; \end{aligned}$$
- Taking $n=3$ in (14) gives
  $$\begin{aligned} &{-}\frac{1}{15}x^{4} + \frac{23}{1890}x^{6} - \frac{41}{37\text{,}800}x^{8} +\frac{53}{831\text{,}600}x^{10} - \frac{74\text{,}677}{27\text{,}243\text{,}216\text{,}000}x^{12} \\ &\quad < \cos x - \biggl(\frac{\sin x}{x} \biggr) ^{ 3} \\ &\quad < -\frac{1}{15} x^{4} +\frac{23}{1890} x^{6} -\frac{41}{37\text{,}800} x^{8} +\frac{53}{831\text{,}600} x^{10} \\ &\quad \quad {}-\frac{74\text{,}677}{27\text{,}243\text{,}216\text{,}000} x^{12} +\frac{989}{10\text{,}897\text{,}286\text{,}400} x^{14}; \end{aligned}$$
- Taking $n=4$ in (14) gives
  $$\begin{aligned} &{-}\frac{x^{4}}{15} + \frac{23 x^{6}}{1890} -\frac{41x^{8}}{37\text{,}800} + \frac{53x^{10}}{831\text{,}600} -\frac{74\text{,}677x^{12}}{27\text{,}243\text{,}216\text{,}000} +\frac{989x^{14}}{10\text{,}897\text{,}286\text{,}400} \\ &\quad \quad {}- \frac{79\text{,}649x^{16}}{33\text{,}345\text{,}696\text{,}384\text{,}000} \\ &\quad < \cos x - \biggl(\frac{\sin x}{x}\biggr) ^{3} \\ &\quad < -\frac{x^{4}}{15} +\frac{23x^{6}}{1890} -\frac{41x^{8}}{37\text{,}800} + \frac{53x^{10}}{831\text{,}600} -\frac{74\text{,}677x^{12}}{27\text{,}243\text{,}216\text{,}000} +\frac{989x^{14}}{10\text{,}897\text{,}286\text{,}400} \\ &\quad \quad {}-\frac{79\text{,}649x^{16}}{33\text{,}345\text{,}696\text{,}384\text{,}000} + \frac{454\text{,}007x^{18}}{8\text{,}869\text{,}955\text{,}238\text{,}144\text{,}000} \end{aligned}$$

etc.

#### Proof of Theorem 1

Consider the function

$$ f(x) = \cos x - \biggl(\frac{\sin x}{x} \biggr) ^{ 3}\quad \mbox{for } x \in \biggl( 0,\frac{\pi }{2}\biggr) . $$

Based on power series expansions (1) and (3), we have

$$ f(x)=\sum_{k=1}^{\infty }{(-1)^{k} A(k) x^{2k}}= \sum_{k=2}^{\infty }{(-1)^{k} A(k) x^{2k}} $$

for all $x \in R$, where

16 $$\begin{aligned} A(k)&=\frac{1}{4}\frac{3^{2k+3}-3}{(2k+3)!} -\frac{1}{(2k)!} =\frac{3^{2k+3}-32k^{3}-96k^{2}-88k-27}{4(2k+3)!}. \end{aligned}$$

For $c=\pi /2$, we have

$$ A(k)>0 \quad \mbox{for } k\geq 2, \quad \mbox{and} \quad \lim_{k\rightarrow \infty }c^{2k}A(k) =0. $$

Also,

$$\begin{aligned}& c^{2}A(k + 1) < A(k) \quad \Longleftrightarrow \quad \\& 32k^{5} + 240k^{4} + \bigl(680 - 8c^{2} \bigr)k^{3} + \bigl(900 - 45c^{2} \bigr)k^{2} + \biggl(548 - {\frac{161c^{2}}{2}} \biggr) k - 45c^{2} + { \frac{477}{4}} \\& \quad < {\frac{3 (4c^{2}k^{2} + 18c^{2}k + 20c ^{2} - 9)}{4}9^{k+1}}. \end{aligned}$$

As the last inequality holds for $k \geq 1$, the assertions of Theorem 1 immediately follow from Proposition 1. □

### Refinements of the inequalities in Statement 2

We propose the following improvement and generalization of Statement 2.

#### Theorem 2

(i) *For every* $x \in ( 0, \frac{\pi }{2}) $ *and every* $n \in N$, *we have*
  17 $$ \sum_{k=2}^{2n} (-1)^{k+1} B(k) x^{2k}< \frac{\sin {x}}{x} -\frac{\cos {x} + 2}{3}< \sum_{k=2}^{2n+1}(-1)^{k+1} B(k) x^{2k}, $$
  *where*
  $$ B(k) = \frac{2}{3}\frac{k - 1}{(2 k + 1)!}. $$
(ii) *For every* $x \in ( 0,\frac{\pi }{2}) $ *and every* $m \in N$, *we have the following error estimation*:
  18 $$ \Biggl\vert \frac{\sin {x}}{x} -\frac{ \cos {x} + 2}{3}-\sum _{k=0}^{m}{(-1)^{k+1}B(k) x^{2k}} \Biggr\vert < B(m+1) x^{2m+2}. $$

#### Examples

Let $x \in ( 0, \frac{\pi }{2}) $.

For $n = 1$, we get Statement 2.

For $n >1 $, we have the following new results:

- Taking $n=2$ in (17) gives

$$\begin{aligned} -\frac{1}{180}x^{4} + \frac{1}{3780}x^{6} -\frac{1}{181\text{,}440}x^{8} & < \frac{\sin x}{x} - \frac{1}{3}\cos x -\frac{2}{3} \\ & < - \frac{1}{180}x^{4} + \frac{1}{3780}x^{6} -\frac{1}{181\text{,}440}x^{8} + \frac{1}{14\text{,}968\text{,}800}x^{10}; \end{aligned}$$

- Taking $n=3$ in (17) gives
  $$\begin{aligned} &{-}\frac{ x^{4}}{180} + \frac{ x^{6}}{3780} - \frac{ x^{6}}{181\text{,}440} + \frac{ x^{10}}{14\text{,}968\text{,}800} - \frac{ x^{12}}{1\text{,}868\text{,}106\text{,}240} \\ &\quad < \frac{\sin x}{x} - \frac{1}{3} \cos x - \frac{2}{3} \\ &\quad < - \frac{ x^{4}}{180} + \frac{ x^{6}}{3780} - \frac{ x^{8}}{181\text{,}440} + \frac{ x^{10}}{14\text{,}968\text{,}800} - \frac{ x^{12}}{1\text{,}868\text{,}106\text{,}240} + \frac{ x^{14}}{326\text{,}918\text{,}592\text{,}000} \end{aligned}$$

etc.

#### Proof of Theorem 2

Consider the function

$$ f(x)= {\frac{\sin x }{x}} - {\frac{1}{3}}\cos x - {\frac{2}{3}} \quad \mbox{for } x \in \biggl( 0, \frac{\pi }{2}\biggr) . $$

Based on power series expansion (1), we have

$$ f(x)=- {\frac{2}{3}} + { \sum_{k=0}^{\infty }}(-1)^{k + 1} { \frac{2}{3}} { \frac{k - 1}{(2 k + 1)!}} x^{2k} = { \sum _{k=2}^{\infty }}(-1)^{k + 1} { \frac{2}{3}} { \frac{k - 1}{(2k + 1)!}} x^{2k}. $$

The sequence $\{ B(k) \} _{k\in N, k \geq 2}$ satisfies the recurrence relation

$$ B(k + 1)= {\frac{k}{2 (k^{2} - 1)(2k + 3)}} B(k). $$

For $c=\pi /2$, we have

$$ B(k)>0 \quad \mbox{for } k\geq 2, \quad \mbox{and} \quad\lim _{k\rightarrow \infty }c^{2k}B(k) =0. $$

Also,

$$\begin{aligned} c^{2}B(k + 1) < B(k) &\quad \Longleftrightarrow\quad \biggl( { \frac{c^{2} k}{2 (k^{2} - 1)(2k + 3)}} - 1 \biggr) \cdot B(k) < 0 \\ &\quad \Longleftrightarrow\quad - { \frac{4k^{3} + 6k^{2} - (c^{2} + 4)k - 6}{2(k^{2} - 1)(2k + 3)}} \cdot B(k) < 0 \\ &\quad \Longleftrightarrow\quad - {\frac{2(2k^{3} - 3)+4 k (k-1) + k (2 k-c^{2})}{2(k^{2} - 1)(2k + 3)}} \cdot B(k) < 0. \end{aligned}$$

As the last inequality holds for every $k \geq 2$, the assertions of Theorem 2 follow from Proposition 1. □

### Refinements of the inequalities in Statement 3

We propose the following improvement and generalization of Statement 3.

#### Theorem 3

(i) *For every* $x \in ( 0, \frac{\pi }{2}) $ *and every* $n \in N$, *we have*
  19 $$\begin{aligned} 3 +\frac{1}{\cos x} \sum_{k=2}^{2n+1}(-1)^{k} C(k) x^{2k} < 2 \frac{\sin {x}}{x} + \frac{\tan x}{x}< 3 +\frac{1}{\cos {x}} \sum_{k=2}^{2n}{ (-1)^{k} C(k) x^{2k}}, \end{aligned}$$
  *where*
  $$\begin{aligned} C(k)= 2\frac{4^{k} - 3 k - 1}{(2k + 1)!}. \end{aligned}$$
(ii) *For every* $x \in ( 0, \frac{\pi }{2}) $ *and every* $m \in N$, $m\geq 2$, *we have the following error estimation*:
  $$ \Biggl\vert 2 \frac{\sin {x}}{x} + \frac{\tan x}{x}- \Biggl( 3 + \frac{1}{\cos x}\sum_{k=2}^{m}{(-1)^{k+1}C(k) x^{2k}} \Biggr) \Biggr\vert < C(m+1)\frac{x^{2m+2}}{\cos x}. $$

#### Examples

Let $x \in ( 0, \frac{\pi }{2}) $.

For $n = 1$, we get Statement 3.

For $n > 1 $, we have the following new results:

- Taking $n=2$ in (19) gives
  $$\begin{aligned} &2 + \frac{1}{\cos x} \biggl( \frac{3}{20}x^{4} - \frac{3}{140}x^{6} + \frac{3}{2240}x^{8} - \frac{1}{19\text{,}800}x^{10}\biggr) \\ &\quad < 2 \frac{\sin x}{x} + \frac{\tan x}{x} \\ &\quad < 2 + \frac{1}{\cos x} \biggl( \frac{3}{20} x^{4} - \frac{3}{140} x^{6} + \frac{3}{2240} x^{8} \biggr) ; \end{aligned}$$
- Taking $n=3$ in (19) gives
  $$\begin{aligned} &2 + \frac{1}{\cos x} \biggl( \frac{3}{20} x^{4} - \frac{3}{140} x^{6} + \frac{3}{2240} x^{8} - \frac{1}{19\text{,}800} x^{10} \\ &\quad \quad {}+ \frac{151}{115\text{,}315\text{,}200} x^{12} - \frac{101}{4\text{,}036\text{,}032\text{,}000} x^{14} \biggr) \\ &\quad < 2 \frac{\sin x}{x} + \frac{\tan x}{x} \\ &\quad < 2 + \frac{1}{\cos x} \biggl( \frac{3}{20} x^{4} - \frac{3}{140} x^{6} + \frac{3}{2240} x^{8} - \frac{1}{19\text{,}800} x^{10} + \frac{151}{115\text{,}315\text{,}200} x^{12} \biggr) \end{aligned}$$

etc.

#### Proof of Theorem 3

Consider the function

$$ f(x) = \frac{\sin {2 x}}{x}+ \frac{\sin {x}}{x} - 3 \cos {x} $$

for $x \in ( 0, \frac{\pi }{2}) $. Based on power series expansion (1), we have

$$\begin{aligned} f(x)&=\sum_{k=0}^{\infty }(-1)^{k} \biggl( { \frac{2^{(2k + 1)}}{(2k + 1) !}} + {\frac{1}{(2k + 1)!}} - { \frac{3}{(2k)!}} \biggr) x^{2k} \\ &=\sum_{k=2}^{\infty } (-1)^{k}{ 2 \frac{4^{k} - 3k - 1}{(2k + 1)!}}x^{2k} \end{aligned}$$

for $x \in ( 0, \frac{\pi }{2}) $. For $c=\pi /2$, we have

$$\begin{aligned} C(k)>0\quad \mbox{for } k\geq 2,\quad \mbox{and} \quad \lim_{k\rightarrow \infty }c^{2k}C(k)=0. \end{aligned}$$

Also,

$$\begin{aligned}& c^{2} C(k + 1) < C(k)\quad \Longleftrightarrow \\& -2 \biggl( 12k^{3} + 38k^{2} + {\frac{(6c^{2} + 76)k}{2}} + 4c^{2} + 12 \biggr) < 4^{k + 1} \bigl( 2(k+c) (k-c) + 5k + 3 \bigr) . \end{aligned}$$

As the last inequality holds for $k \geq 2$, the assertions of Theorem 2 immediately follow from Proposition 1. □

### Refinements of the inequalities in Statement 4

We propose the following improvement and generalization of Statement 4.

#### Theorem 4

(i) *For every* $x \in ( 0, \frac{\pi }{2}) $ *and every* $n \in N$, *we have*
  20 $$\begin{aligned} 2 + \frac{1}{\cos x}\sum_{k=2}^{2n+1} (-1)^{k} D(k) x^{2k} &< \biggl( \frac{ \sin {x}}{x} \biggr) ^{ 2} + \frac{\tan {x}}{x} \\ &< 2 + \frac{1}{\cos x} \sum_{k=2}^{2n}(-1)^{k} D(k) x^{2k}, \end{aligned}$$
  *where*
  $$\begin{aligned} D(k) = { \frac{1}{4}}{ \frac{ - 9 + 3^{2k + 2} - 40k - 32k^{2}}{(2k + 2)!}}. \end{aligned}$$
(ii) *For every* $x \in ( 0, \frac{\pi }{2}) $ *and every* $m \in N, m\geq 2$, *we have the following error estimation*:
  $$ \Biggl\vert \biggl( \frac{\sin {x}}{x} \biggr) ^{ 2} + \frac{\tan {x}}{x} - \Biggl( 2+\frac{1}{\cos x}\sum _{k=2}^{m}{(-1)^{k+1}D(k) x^{2k}} \Biggr) \Biggr\vert < D(m+1)\frac{x^{2m+2}}{\cos x}. $$

#### Examples

Let $x \in ( 0,\frac{\pi }{2}) $.

For $n = 1$, we get Statement 4.

For $n > 1$, we have the following new results:

- Taking $n=2$ in (20) gives
  $$\begin{aligned} &2 +\frac{1}{\cos x} \biggl(\frac{8}{45} x^{4} - \frac{4}{105} x^{6} +\frac{19}{4725} x^{8} - \frac{37}{133\text{,}650} x^{10} \biggr) \\ &\quad < \biggl( \frac{\sin x}{x} \biggr) ^{ 2} + \frac{\tan x}{x} \\ &\quad < 2 + \frac{1}{\cos x} \biggl( \frac{8}{45} x^{4} - \frac{4}{105} x^{6} + \frac{19}{4725} x^{8} \biggr); \end{aligned}$$
- Taking $n=3$ in (20) gives
  $$\begin{aligned} &2 +\frac{1}{\cos x} \biggl(\frac{8}{45} x^{4} - \frac{4}{105} x^{6} + \frac{19}{4725} x^{8} - \frac{37}{133\text{,}650} x^{10} \\ &\quad \quad {}+ \frac{283}{20\text{,}638\text{,}800} x^{12} - \frac{3503}{6\text{,}810\text{,}804\text{,}000} x^{14} \biggr) \\ &\quad < \biggl( \frac{\sin x}{x} \biggr) ^{ 2} + \frac{\tan x}{x} \\ &\quad < 2 + \frac{1}{\cos x} \biggl( \frac{8}{45} x^{4} - \frac{4}{105} x^{6} + \frac{19}{4725} x^{8} \\&\quad \quad {}- \frac{37}{133\text{,}650} x^{10} + \frac{283}{20\text{,}638\text{,}800} x^{12} \biggr) \end{aligned}$$

etc.

#### Proof of Theorem 4

Consider the function

$$ f(x)={\frac{\cos x-\cos ^{3}x}{x^{2}}}+\frac{\sin x}{x}-2\cos x \quad \mbox{for } x \in \biggl( 0, \frac{\pi }{2}\biggr) . $$

Based on power series expansion (2), we have

$$\begin{aligned} f(x) &= {\sum_{k=0}^{\infty }}(-1)^{k} \biggl( - {\frac{1}{(2k + 2)!} } + {\frac{1}{4}} {\frac{3^{(2k + 2)} + 3}{ (2k + 2)!}} + {\frac{1}{(2k + 1)!}} - {\frac{2}{(2k)!}} \biggr) x^{2k} \\ &= {\sum_{k=0}^{\infty }}(-1)^{k} { \frac{ 9^{k + 1} - (32k^{2} + 40k +9)}{4 (2k + 2)!}}x^{2k} \\ &= \sum_{k=2}^{\infty }(-1)^{k}{\frac{9^{k + 1} - (32k^{2} + 40k +9) }{4 (2k + 2)!}}x^{2k}. \end{aligned}$$

For $c=\pi /2$, we have

$$ D(k)>0 \quad \mbox{for } k\geq 2,\quad \mbox{and} \quad \lim_{k\rightarrow \infty }c^{2k}D(k) =0. $$

Also,

$$\begin{aligned} &c^{2} D(k + 1) < D(k) \quad \Longleftrightarrow \\ &32k^{4} + 152k^{3} + \bigl( 245- 8 c^{2} \bigr)k^{2} + \biggl( {\frac{303}{2}} - 26c^{2}\biggr) k + 27 - {\frac{81c^{2}}{4}} \\ &\quad < \frac{1}{4}{9^{k+1}\bigl( 4 k^{2} + 14k + 12- 81c^{2} \bigr)}. \end{aligned}$$

As the last inequality holds for $k \geq 2$, the assertions of Theorem 2 immediately follow from Proposition 1. □

### Refinements of the inequalities in Statement 5

We prove the following generalization of Statement 5.

#### Theorem 5

*For every* $x \in ( 0, \frac{\pi }{2}) $ *and* $m \in N$, $m \ge 2$, *the following inequalities hold*:

21 $$\begin{aligned} &2 + \sum_{k = 2}^{m - 1}\frac{{\vert {{\boldsymbol{B} _{2k}}} \vert (2k - 2){4^{k}}}}{{(2k)!}}{x^{2k}} + { \biggl( {\frac{ {2x}}{\pi }} \biggr) ^{ 2n}} \Biggl( { \frac{{{\pi^{2}}}}{4} - 2 - \sum _{k = 2}^{m - 1} \frac{{\vert {{\boldsymbol{B}_{2k}}} \vert (2k - 2){4^{k}}}}{{(2k)!}}{{ \biggl( { \frac{\pi }{2}} \biggr) }^{2k}}} \Biggr) \\ &\quad > { \biggl( {\frac{x}{{\sin x}}} \biggr) ^{2}} +\frac{x}{{\tan x}} \\ &\quad > 2 + \sum_{k = 2}^{m} \frac{{\vert {{\boldsymbol{B}_{2k}}} \vert (2k - 2){4^{k}}}}{{(2k)!}}{x^{2k}}, \end{aligned}$$

*where* $\boldsymbol{B}_{i}$ *are Bernoulli’s numbers*.

#### Proof of Theorem 5

Consider the function

$$ f(x)=\biggl( \frac{x}{\sin x} \biggr) ^{2} + \frac{x}{\tan x} \quad \mbox{for } x \in \biggl( 0, \frac{\pi }{2}\biggr) . $$

Based on series expansion (5) and (6), we have

$$\begin{aligned} f(x) &={x^{2}} \Biggl( {\frac{1}{{{x^{2}}}} +\sum _{k = 1}^{ \infty } \frac{{\vert {{\boldsymbol{B} _{2k}}} \vert (2k - 1){4^{k}}}}{{(2k)!}}{x^{2k - 2}}} \Biggr) + x \Biggl( {\frac{1}{x} - \sum_{k = 1}^{ \infty } \frac{ {\vert {{\boldsymbol{B}_{2k}}} \vert {4^{k}}}}{{(2k)!}}{x^{2k - 1}}} \Biggr) \\ &=2 + \sum_{k = 1}^{ \infty } \frac{{\vert {{\boldsymbol{B} _{2k}}} \vert (2k - 1){4^{k}} - {4^{k}}}}{{(2k)!}}{x^{2k}} \\ &=2 + \sum_{k = 2}^{ \infty } \frac{{\vert {{\boldsymbol{B} _{2k}}} \vert (2k - 2){4^{k}}}}{{(2k)!}}{x^{2k}} \\ &>2 + \sum_{k = 2}^{m} \frac{{\vert {{\boldsymbol{B}_{2k}}} \vert (2k - 2){4^{k}}}}{{(2k)!}}{x^{2k}}. \end{aligned}$$

Since all coefficients are positive, by applying Theorem WD, we get the inequalities in the statement of the theorem. □

#### Examples

For $x \in ( {0, \frac{\pi }{2} } ) $ and $f(x) = { (\frac{x}{{\sin x}} ) ^{ 2}} + \frac{x}{{\tan x}}$, we show the inequalities for $m = 2, 3, 4, 5$.

- For $m=2$:
  $$\begin{aligned} 2+{ \biggl( { \frac{2}{\pi } } \biggr) ^{ 4}} \biggl( -2 + \frac{{{\pi^{2}}}}{4} \biggr) {x^{4}} > f(x) > 2+\frac{2}{{45}} {x^{4}}. \end{aligned}$$
  On the right-hand side we see the inequality from Statement 5.
- For $m = 3$:
  $$\begin{aligned} 2+ \frac{2}{{45}} {x^{4}}+ { \biggl( {\frac{2}{\pi } } \biggr) ^{ 6}} \biggl(-2 + \frac{{{\pi^{2}}}}{4} - \frac{{{\pi^{4}}}}{{360}} \biggr) {x^{6}}> f(x) >2+ \frac{2}{{45}} {x^{4}}+ \frac{8}{{945}} {x^{6}}. \end{aligned}$$
- For $m = 4$:
  $$\begin{aligned} &2 + \frac{2}{{45}} {x^{4}} + \frac{8}{{945}} {x^{6}} + { \biggl( \frac{2}{\pi } \biggr) ^{ 8}} \biggl( -2 + \frac{{{\pi^{2}}}}{4} - \frac{{{\pi^{4}}}}{{360}} - \frac{{{\pi^{6}}}}{{7560}} \biggr) {x^{8}} \\ &\quad >f(x)> 2 + \frac{2}{{45}} {x^{4}} + \frac{8}{{945}} {x^{6}} + \frac{2}{{1575}} {x^{8}}. \end{aligned}$$
- For $m = 5$:
  $$\begin{aligned} &2 +\frac{2}{{45}} {x^{4}} + \frac{8}{{945}} {x^{6}} + \frac{2}{{1575}} {x^{8}} + { \biggl( \frac{2}{\pi } \biggr) ^{ 10}} \biggl( - 2 + \frac{{{\pi^{2}}}}{4} - \frac{{{\pi^{4}}}}{{360}} - \frac{{{\pi^{6}}}}{{7560}} - \frac{{{\pi^{8}}}}{{201\text{,}600}} \biggr) {x^{10}} \\ &\quad > f(x) > 2 + \frac{2}{{45}} {x^{4}} + \frac{8}{{945}} {x^{6}} + \frac{2}{{1575}} {x^{8}} + \frac{{16}}{{93\text{,}555}} {x^{10}}. \end{aligned}$$

#### Remark

Let us notice that Theorem WD allows for the approximation error to be estimated. The difference between the right-hand side and the left-hand side of the double inequality in Theorem 5 can be represented by the following function:

$$ {R_{n}} ( x ) = \Biggl( {f \biggl( {\frac{\pi }{2}} \biggr) - 2 - \sum_{k = 1}^{n} \frac{{\vert {{\boldsymbol{B}_{2k}}} \vert (2k - 2){4^{k}}}}{{(2k)!}}{{ \biggl( {\frac{\pi }{2}} \biggr) }^{2k}}} \Biggr) { \biggl( { \frac{{2x}}{\pi }} \biggr) ^{ 2n }}. $$

The maximum values of $R_{n}(x)$ are reached at $\frac{\pi }{2}$, and their values for $n=3, 4, 5$ and 6 are $6.97 \times 10^{-2}$, $2.26 \times 10^{-2}$, $6.95 \times 10^{-3}$, and $2.06 \times 10^{-3}$, respectively.

### Refinements of the inequalities in Statement 6

We propose the following generalization of Statement 6.

#### Theorem 6

*For every* $x \in ( {0, \frac{\pi }{2}} ) $ *and* $m \in N$, $m \ge 3$, *the following inequality holds*:

22 $$\begin{aligned} &4 + \sum_{k = 1}^{m - 1} \frac{3{\vert {{\boldsymbol{B}_{2k}}} \vert ({2^{2k}} - 2) + {{(-1)}^{k}}}}{{(2k)!}}{x^{2k}} \end{aligned}$$

23 $$\begin{aligned} &\quad \quad {}+ {{{ \biggl(\frac{2x}{\pi } \biggr) ^{ 2m}}}} \Biggl({f \biggl( \frac{\pi }{2} \biggr) - 4 - \sum _{k = 1}^{m - 1} \frac{3{\vert {{\boldsymbol{B}_{2k}}}\vert ( {2^{2k}} - 2) + {{( - 1)}^{k}}}}{{(2k)!}}{{ \biggl( { \frac{ \pi }{2}} \biggr) }^{ 2k}}} \Biggr) \end{aligned}$$

24 $$\begin{aligned} &\quad >3\frac{x}{{\sin x}} + \cos x > 4 + \sum_{k = 1} ^{m} \frac{{3\vert {{\boldsymbol{B}_{2k}}} \vert ({2^{2k}} - 2) + {{( - 1)}^{k}}}}{{(2k)!}}{x^{2k}}, \end{aligned}$$

*where* $\boldsymbol{B}_{i}$ *are Bernoulli’s numbers*.

#### Proof of Theorem 6

Consider the function

$$ f(x)=3\frac{x}{{\sin x}} + \cos x \quad \mbox{for } x \in \biggl( 0, \frac{\pi }{2} \biggr) . $$

Based on the series expansion (1) and (4), we have

$$ f(x)= 4 + \sum_{k = 1}^{ \infty } \frac{{3\vert {{\boldsymbol{B} _{2k}}} \vert ({2^{2k}} - 2) + {{( - 1)}^{k}}}}{{(2k)!}}{x^{2k}}. $$

It is easy to verify that $3\vert {{\boldsymbol{B}_{2k}}} \vert ( {2^{2k}} - 2) > 1$ for $k\geq 2$, and that it is equal to 1 for $k = 1$, therefore all corresponding coefficients are positive. Now, using Theorem WD, we get the inequalities in the statement of the theorem. □

#### Examples

For $x \in ( 0, \frac{\pi }{2}) $ and $f(x)=3\frac{x}{{\sin x}} + \cos x$, we show the inequalities for $m = 3, 4, 5, 6$.

- For $m = 3$:
  $$\begin{aligned} 4+ \frac{1}{{10}} {x^{4}} + { \biggl( { \frac{2}{\pi } } \biggr) ^{ 6}} \biggl( - 4 + \frac{{3\pi }}{2} - \frac{{{\pi^{4}}}}{{160}} \biggr) {x^{6}} > f(x) > 4 + \frac{1}{{10}} {x^{4}} + \frac{1}{{210}} {x^{6}}. \end{aligned}$$
  On the right-hand side we see the inequality from Statement 6.
- For $m = 4$:
  $$\begin{aligned} &4 + \frac{1}{{10}} {x^{4}} + \frac{1}{{210}} {x^{6}} + { \biggl( { \frac{2}{\pi } } \biggr) ^{ 8}} \biggl( -4 + \frac{{3\pi }}{2} - \frac{{{\pi^{4}}}}{{160}} - \frac{{{\pi^{6}}}}{{13\text{,}440}} \biggr) {x^{8}} \\ &\quad > f(x) > 4 + \frac{1}{{10}} {x^{4}} + \frac{1}{{210}} {x^{6}} + \frac{{11}}{{16\text{,}800}} {x^{8}}. \end{aligned}$$
- For $m = 5$:
  $$\begin{aligned} &4 + \frac{1}{10} x^{4}+\frac{1}{210} x^{6} + \frac{11}{16\text{,}800} x^{8} \\ &\quad \quad {}+\biggl(\frac{2}{\pi } \biggr) ^{10} \biggl(- 4+ \frac{3\pi }{2}-\frac{\pi^{4}}{160}-\frac{\pi^{6}}{13\text{,}440}-\frac{11\pi^{8}}{4\text{,}300\text{,}800} \biggr) x^{10} \\ &\quad > f(x) > 4+\frac{1}{10} {x^{4}}+\frac{1}{210} x^{6}+\frac{11}{16\text{,}800} {x^{8}}+\frac{53}{831\text{,}600} x^{10}. \end{aligned}$$
- For $m = 6$:
  $$\begin{aligned} &4 + \frac{x^{4}}{10} +\frac{x^{6}}{210} +\frac{11x^{8}}{16\text{,}800} + \frac{53x^{10}}{831\text{,}600} \\ &\quad \quad {}+\biggl( \frac{2}{\pi}\biggr)^{ 12} \biggl(-4 + \frac{3\pi }{2} -\frac{\pi^{4}}{160} -\frac{\pi^{6}}{13\text{,}440} -\frac{11\pi^{8}}{4\text{,}300\text{,}800} -\frac{53\pi^{10}}{851\text{,}558\text{,}400}\biggr)x^{12} \\ &\quad > f(x) > 4+\frac{x^{4}}{10}+\frac{x^{6}}{210}+\frac{11x^{8}}{16\text{,}800}+ \frac{53x^{10}}{831\text{,}600}+\frac{117\text{,}911x^{12}}{18\text{,}162\text{,}144\text{,}000} . \end{aligned}$$

#### Remark

The difference between the right-hand side and the left-hand side of the double inequality in Theorem 6 can be represented by the following function:

$$ {R_{n}} ( x )= \Biggl( {f \biggl( {\frac{\pi }{2}} \biggr) - 4 + \sum_{k = 1}^{n} \frac{{3\vert {{\boldsymbol{B}_{2k}}} \vert ({2^{2k}} - 2) + {{( - 1)}^{k}}}}{{(2k)!}}{{ \biggl( {\frac{\pi }{2}} \biggr) }^{2k}}} \Biggr) { \biggl( { \frac{{2x}}{\pi }} \biggr) ^{ 2n}}. $$

The maximum values of $R_{n}(x)$ are reached at $\frac{\pi }{2}$, and their values for $n=3,4,5$, and 6 are $3.20\times 10^{-2}$, $7.78\times 10^{-3}$, $1.95\times 10^{-3}$, and $4.88\times 10^{-4}$, respectively.

## Conclusion

The idea to compare and replace functions with their corresponding power series to get more accurate approximations was used in [9, 10], and [7]. Following the same idea, in this paper we extended the natural approach. We proposed and proved new inequalities which represent refinements and generalizations of the inequalities stated in [2], related to Wilker–Cusa–Huygens’s inequalities.

Note that proofs of the new inequalities (14), (17), (19), (20), (21), and (22) for any fixed $n, m \in N$ can be obtained by substituting $x = \sin t$ for $t \in [ 0, \frac{\pi }{2}] $ and using the methods and algorithms developed in [11] and [12]. However, our approach provides proofs for the approximation of the corresponding function by the inequality of an arbitrary degree.

The results of the present research can be used to verify as well as to refine a broad category of inequalities. For example, Bercu ([13], Theorem 2.4) proved the following inequalities:

$$\begin{aligned} & \biggl(\frac{x}{\sin x} \biggr) ^{2} +\frac{x}{\tan x} \\ &\quad > \frac{11\text{,}220 x^{10} - 205\text{,}560x^{8} -14\text{,}256\text{,}000x^{6} + 512\text{,}179\text{,}200x^{4} - 3\text{,}157\text{,}056\text{,}000x^{2} + 13\text{,}716\text{,}864\text{,}000}{242x^{12} - 8580x^{10} + 25\text{,}560x^{8} - 1\text{,}080\text{,}000x^{6} + 103\text{,}680\text{,}000x^{4} - 1\text{,}578\text{,}528\text{,}000x^{2} + 6\text{,}858\text{,}432\text{,}000} \\ &\quad > 2 +\frac{2}{45}x^{4} > 2 \end{aligned}$$

for every $x \in (0, b)$, where $b=\sqrt{\frac{240-6\sqrt{1090}}{17}} = 1.5701 \ldots <\frac{\pi }{2}$.

According to Theorem 5, taking $m=7$ in (21) gives

$$ \biggl(\frac{x}{\sin x} \biggr) ^{ 2} +\frac{x}{\tan x} > P_{7}(x) \quad \mbox{for } x \in \biggl( 0, \frac{\pi }{2} \biggr) , $$

where

$$ P_{7}(x)= 2 + \sum_{k = 2}^{7} \frac{{\vert {{\boldsymbol{B}_{2k}}} \vert (2k - 2){4^{k}}}}{{(2k)!}}{x^{2k}}. $$

Thus Bercu’s inequality is reduced to the following decidable problem (see [14, 15]): for every $x \in ( 0, \frac{\pi }{2} ) $, it is true that

$$\begin{aligned} &P_{7}(x) \\ &\quad > \frac{11\text{,}220 x^{10} - 205\text{,}560 ,x^{8} - 14\text{,}256\text{,}000 x^{6} + 512\text{,}179\text{,}200 x^{4} - 3\text{,}157\text{,}056\text{,}000 x^{2} + 13\text{,}716\text{,}864\text{,}000}{ 242 x^{12} - 8580 x^{10} + 25\text{,}560 x^{8} - 1\text{,}080\text{,}000 x^{6} + 103\text{,}680\text{,}000 x^{4} - 1\text{,}578\text{,}528\text{,}000 x^{2} + 6\text{,}858\text{,}432\text{,}000 }. \end{aligned}$$

The above inequality is a refinement of the inequality obtained by Bercu. Moreover, the above inequality shows that Bercu’s inequality holds true over an extended interval $( 0, \frac{\pi }{2}) $.

Similarly, Theorems 1, 2, 3, 4, 5, and 6 can be applied to other results and inequalities from [13, 16], as well as to a broad category of analytical inequalities.
